# Exploring How Ethnoculturally Diverse Surgical Patients and Families Perceive and Deal with Pain Before Hospital Admissions in Ethiopia: Qualitative Descriptive Study

**DOI:** 10.3390/healthcare13101142

**Published:** 2025-05-14

**Authors:** Getu Ataro Hanago, Matthias Siebeck, Samuel Jilo Dira, Tefera Tadesse, Dominik Irnich

**Affiliations:** 1 College of Medicine and Health Sciences, Hawassa University, Hawassa P.O. Box 1560, Ethiopia; 2 Institute of Medical Education, LMU University Hospital, LMU Munich, 80336 Munich, Germany; 3 Department of Anthropology, Hawassa University, Hawassa P.O. Box 1560, Ethiopia; 4 Educational Development and Quality Center, University of Global Health Equity, Kigali P.O. Box 6955, Rwanda; ttadesse@ughe.org; 5 Multidiciplinary Pain Center, Department of Anesthesiology, LMU University Hospital, LMU Munich, 80336 Munich, Germany

**Keywords:** pain, perception of pain, cultural competence, traditional medicine

## Abstract

Background: Pain is one of the major medical and public health challenges in the world, and its prevalence is unaffected by some ‘painful’ pandemics of the past, reflecting the deep-rooted causes of other origins. Surgical conditions accounting for one-third of the global burden of disease are associated with physical pain, either as a symptom or complication. For effective perioperative pain management of culturally diverse patients, it is imperative to understand how patients view and deal with pain. Therefore, this study explored study participants’ pain experience as well as their perception of the causes, consequences, and treatment options of surgical condition-related pain. Methods: With a subjectivist research paradigm, as well as relativist and interpretivist ontological and epistemological underpinnings, a qualitative description study design was used to interview 11 patients with abdominal surgical conditions and 12 family members taking care of those patients in three hospitals. Following inductive coding, thematic analysis was employed, which resulted in four themes. Findings: Patients and their families shared various experiences and perceptions of the meanings, causes, consequences, and treatment options of pain, summarized under four emergent themes: *Perception of pain meaning*, *causes*, *and consequences*; *sustenance for pain relief*; *traditional pain relievers*; *and conventional pain medicine*. Conclusions: This study highlighted that ethnoculturally diverse surgical patients and their families may have unique perceptions of pain and use various treatment approaches at home, which might have implications on perioperative pain management. Therefore, professionals at the participating hospitals and elsewhere with similar contexts should consider these cultural phenomena during surgical pain management.

## 1. Introduction

Pain has remained one of the major medical and public health problems in the world. Globally, 34.1% of the population was estimated to experience physical pain in 2022, and this magnitude was almost stable throughout pandemics of the past, suggesting deep-rooted causes of other origins [1]. Surgical diseases account for 30% of the global burden of disease [2] and 28% of the overall disease burden in low- and middle-income countries, which is related to surgical conditions, may also be associated with chronic pain [3]. Surgical patients can present with various degrees of acute or chronic pain, detected during a preoperative evaluation [4]. Based on their respective ethnicity-based cultural (ethnocultural) backgrounds, patients and their families may distinctly perceive and deal with the pain of surgical conditions [5]. Culture is a multiplex social construct that shapes the behaviors of individuals in a society [6]. Similar to concepts of culture and ethnicity, pain is also a complex phenomenon [7,8] that needs to be supported with theoretical frameworks for a better understanding of the context.

Numerous theories and models have been used to explain the mechanisms of pain [9]. Aside from their application in clinical discourses, these theories and models can have several roles in qualitative pain studies. Theories and models are important for understanding phenomena, guiding epistemological and methodological approaches, and facilitating the interpretation of the findings [10]. The biopsychosocial (BPS) model is considered a more comprehensive and widely accepted model than any other pain theory or model [11,12], and it has played a role in informing inquiry and framing the discussion of the current study. The BPS model promotes a holistic approach to the assessment and management of pain, taking into account the interaction of the body, mind, and sociocultural aspects of patients to explain their pain experiences [13,14,15]. Historically, it was developed to bridge gaps between somatosensory (e.g., specificity and pattern) and psychosomatic (e.g., gate-controlled and neuromatrix) pain theories in explaining important aspects of pain [16]. Despite its wider applicability in clinical practices and research endeavors, the model is not free from criticism, such as for lacking scientific rigor as a theory or model per se, and is known to inappropriately fragment patients’ pain into three distinct components without deeper reflection on the inter-componental linkage [17,18,19]. However, we believe that the apparent strengths of the model outweigh the postulated downsides, and we found the model appropriate for the purpose of our study.

A great deal of literature revealed that ethnoculturally diverse patients perceive the meaning and causes of pain, hence, practice their treatment options differently. For instance, African-Americans and Caucasians are more likely to attach negative meanings to pain than Asians and Hispanics [20]. In some Asian cultures, the meaning and causes of pain are related to culture and religion; it is generally viewed as a means of spiritual purification, personal maturity, and growth [21]. Herbal medicine, non-herbal traditional medicine, and other traditional and religious practices are also used to alleviate pain among people from different cultures [22,23,24,25]. We argue that these practices are not uniform across the regions and countries of the world. For perioperative prevention and treatment of surgical pain, it is imperative to have a clear image of how people make sense of and deal with pain before hospital admissions. Therefore, aimed at answering the research question, “How do surgical patients and their families perceive and alleviate physical pain of surgical conditions?”, the primary objective of the current study was to explore the perceptions and treatment options of pain practiced at home by surgical patients and their families, who were selected from hospitals of three ethnic-based regions of Ethiopia.

## 2. Materials and Methods

### 2.1. Research Paradigm and Design

We employed a qualitative description study design [26] based on our ontological and epistemological assumptions. The methodology of the current study was informed by a subjectivist research paradigm with relativism and interpretivism as ontological and epistemological underpinnings. Disclosing the paradigm and related perspectives, i.e., the worldview or theory-informed assumptions towards reality, knowledge, human interactions, and related processes, it is important to unfold how the researchers approached these phenomena, selected the methodology, determined the design, interpreted the results, and drew specific conclusions [27,28,29]. Four commonly discussed research paradigms have been dominant in the field of medical science and education research: positivism, post-positivism, interpretivism/constructivism, and critical theory [30]. Ontology, epistemology, and methodology are the three most common pillars of any of these research paradigms [31]. Yet, qualitative authors have not reached a consensus about the distinction between ontology and epistemology, unlike their agreement about the definitions of the same concepts [32]. Therefore, it is not uncommon to see authors using features of ontology, epistemology, and the overall paradigm interchangeably. An evaluation of our ontological assumptions, as well as our understanding of the nature of the research question, and utilizing our previous experience, helped us to pick our paradigm of choice. Accordingly, we believe that the meaning of pain and concepts of its treatments are constructed in culturally diverse communities, and we also cannot completely ignore our own values and experiences while collecting, analyzing, and interpreting the data.

### 2.2. Study Setting

The current study has grown out of an ongoing project where we examine the ethnocultural determinants of surgical pain management, based on qualitative and quantitative sets of data, earlier work of which, following a different qualitative approach and area of focus for the same participants (Table 1), was published elsewhere [33]. This study was conducted in three hospitals found in three different ethnic-based regions of Ethiopia, namely, Sidama Region, Oromia Region, and Central Ethiopia Region, from October 2022 to April 2023. Oromia, being the largest region of the country, shares common geographical boundaries with the other two regions, and its people (Oromo) are expected to share some elements of culture with their Gurage and Sidama counterparts due to cultural diffusion. From the selected hospitals, eleven patients and twelve patients’ family members were purposively recruited for the interviews and focused discussions. Whereas all patients participated in the in-depth interviews, patients’ family members exclusively took part in the two focus group discussions (FGDs). Three in-depth interviews and one FGD were conducted at Hawassa University Teaching Hospital in the Sidama region, whereas four in-depth interviews and one FGD were conducted at Butajira General Hospital in the Central Ethiopia region. The remaining four in-depth interviews were conducted in the Shashemene Referral Hospital in the Oromia region. As a result of a decreased number of patients admitted for elective intrabdominal surgeries in the hospitals due to campaigns of treating civil war casualties in the hospitals, the data collection took a total of seven months.

### 2.3. Participants

Participation in this study was voluntary, and verbal informed consent was taken before each interview or focused discussion from the participants themselves, as they were all adults and capable of giving consent. The consent was documented with audio records and witnessed by participants’ family members who did not participate in the study. Consent includes the publication of anonymous quotes along with the study output. The participants were informed that their privacy and anonymity would be maintained throughout the study, and their participation would not have any effect on the course of their treatment. All patients underwent abdominal surgery, whereas family members took care of the patients. Moreover, all patients had pain as one of their symptoms of their surgical conditions. The details of the basic characteristics of the study participants are presented in Table 1. Several intrabdominal surgical conditions cause pain [34], which justifies the selection of patients with abdominal surgical conditions and their family members in this study. During data collection, two patients were excluded from the interview because one patient developed severe postoperative complications for which an emergency relaparotomy was planned, whereas the other one developed intolerable postoperative pain at the time of the interview, for which he refused to participate.

### 2.4. Procedures of Data Collection and Analysis

The interviews and focused discussions were conducted face-to-face postoperatively in local languages by the first author with the assistance of translators who were fluent in two or more languages. In the context of Ethiopia, a specific language can predominantly be spoken by an ethnic group and serve as a base for constructing a group identity [35]. Purposive sampling technique was used to recruit patients who underwent abdominal surgeries and their families with ethnocultural background of interest in each study site. The duration of each in-depth interview and FGD was 20–30 min and 40–50 min, respectively. Each participant was contacted and interviewed within three days after surgery, when the patients were assumed to have recovered from the acute effects of anesthesia and surgery, as evaluated by their respective care providers. Semi-structured questions were used to guide the interviews and discussions. We conducted interviews on the bedsides of surgical wards where the patients received postoperative care, whereas FGDs were carried out in the hospital, outside of the wards. In addition to questions about socio-demographics, other personal information, probing questions, and examples of the main interview and discussion items included the following: *1. Why do you think you feel pain? 2. How do individuals understand pain in your culture? 3. How do individuals respond to pain in your culture? How about you? 4. What do you do to get pain relief before surgery or at home?* The interviews were audio-recorded, and the recordings were evaluated by a researcher to check for the point where no new ideas emerged, known as a saturation point. As suggested by the literature on qualitative studies [36,37], the saturation point is used to determine a sample size—i.e., in our case, 11 patients (6 females and 5 males) and 12 family members (6 females and 6 males). The sample size determination was consistent with a report by Hagaman and Wutich [38], where fewer than 16 interviews had been suggested to be sufficient for identifying common themes in cross-cultural studies. To enhance the credibility and validity of the findings, as well as the richness of the data [39,40], we triangulated our data sources by integrating in-depth interviews, FGDs, and field notes for the same topic. Based on the general inductive approach of analysis [41], audio records were transcribed verbatim, and the transcripts were read repeatedly by a researcher to identify categories and themes. More specifically, transcribed texts were prepared and cleaned in the word processor, raw data were read to obtain an overview, the raw data were closely read again, categories and themes were created, texts were coded in multiple categories as well as uncoded texts being identified, and eventually the themes were revised and refined to produce the final themes of the findings with relevant quotes. Thematic analysis was conducted by the lead researcher; however, the emerging themes were shared among all researchers for further discussion, aimed at obtaining more insights and refining themes and the overall findings. We carried out peer debriefing as an additional means of increasing the credibility of the findings. The Standard for Reporting Qualitative Research (SRQR) [42] was used as a reporting tool (Table S1).

### 2.5. Reflexivity

The practice of reflecting on a researcher’s positionality throughout the stages of study plays a crucial role in showcasing the researcher’s subjectivity and improving the credibility of the qualitative findings [43]. It also helps readers to extract the best possible interpretation from the reported findings. Reflecting on the position of the primary investigator of this study, he can be considered an insider to one of the ethnocultures and, to some extent, familiar with the other two. Accordingly, views about pain, pain-relieving approaches, use of traditional medicine, and the perceived effectiveness of available treatment options are different among the three groups. Also, the investigator’s experience as an ethnocultural group member was not beyond mild physical pain due to toothaches, abdominal cramps, and painful minor eye trauma treated with herbal medicines. In settings where Western medicine is promoted as an orthodox treatment approach, it is also not uncommon to preoperatively encounter rural patients who used traditional medicines and other unprescribed pharmacologic agents for pain relief. However, a special meaning ascribed to pain was a new insight. Based on personal experiences of research and professional practice in culturally diverse settings, the cultural dimension, in addition to the biomedical views of the etiology, assessment, and treatment of pain, has recently been acknowledged. Our recent qualitative work also demonstrated how expressing pain is experienced in different cultures and gave us clues that there might be ethnocultural differences in the meaning and treatment options of pain. In recent qualitative works, some of the authors opted to hold a subjectivist and interpretivist worldview [33,44]. However, we believe that positionality is not something researchers hold once and for all, and our position has somehow adjusted in this piece of work and moved further to the relativist ontological and interpretivist epistemological position, and, ultimately, we chose a qualitative descriptive study design as an underpinning approach of this study. Apart from our research philosophical positions, we are also aware that the BPS model, which we adopted to frame our discussions, may be influenced by a hybrid realism and constructionism worldview when looked deeper into its philosophical foundation [45]. However, we deliberately disregarded the nuances of its philosophical assumptions and have focused only on the body–mind–culture aspects of its clinical application. Potential influences related to the discussed positionality are worth taking into account while interpreting the findings of the current study.

## 3. Findings

A total of 23 patients and their family members completed this study. The age of the respondents ranges from 17 years to 75 years. All respondents had a recent or past experience of pain, for which they or their family had some opinion and sought some pain relief. Our analysis resulted in four main themes: *perception of pain meaning*, *causes*, *and consequences*; *sustenance for pain relief*; *traditional pain relievers*; and *conventional pain medicine* (Table 2). In the following sections, we will describe the themes in detail, along with the example texts quoted from the transcript.

### 3.1. Theme 1: Perception of Pain’s Meaning, Causes, and Consequences

A multitude of insights emerged regarding how participants understand pain and why they thought they felt it, from the perspectives of their respective ethnocultural backgrounds. Generally, it was perceived that the possible causes of pain could be the underlying surgical diseases, surgical wounds, nonspecific ailments believed to exist in the community, punishment for one’s bad deeds, the will of God or Allah, or the effects of evil spirits. The underlying disease, the surgical wound, and the increased temperature around the wound were believed to be the reasons for the pain: “It was because of the [gallbladder] stone previously; and it is due to the wound now. The wound is getting warm and that’s why there is pain. Body gets warm and causes pain at the wound …” (Patient #4; 60yo). Some respondents believed that the operation was the reason for their pain and disregarded other causes: “It is because of the operation … my body was touched (cut with surgical knife), … The pain could be due to these reasons …”(Patient #8; 42yo). Supernatural forces or entities—either God/Allah or an evil spirit—were also believed to be reasons for pain: “Allah brought it and Alah can get rid of it. Yes; it’s related to that” (Patient #5; 39yo); “… I also believe that God allows pain to happen” (FGD1 Participant #4; 17yo). Within the community where the respondents belong, “Some people say ‘pain is something related to evil spirit’ …” (Patient #2; 75yo). Pain was also believed to be related to one’s bad deeds: “It is something (related to) a person’s bad deeds that results in the pain of the person …” (FGD1 Participant #5; 35yo). Moreover, some respondents also related pain to ailments that they traditionally believed to exist in their respective community: “They say pain is due to ‘*Offe*’ (to mean something swollen in Gurage language) … That causes pain” (Patient #5; 39yo). As is the case among most Ethiopians, “Pain is assumed to be due to ‘cold’ …” (Patient #10; 45yo). Almost in every part of Ethiopia, ‘*Cold*’ is a construct used to describe a broad range of diseases and ill-health manifested with all or part of the clinical signs and symptoms, such as body pain, headache, fever, cough, etc.

Regardless of its cause, pain was believed to have various unwanted consequences, ranging from simple bodily harm to claiming the lives of individuals unless treated in a timely manner:

“Pain is harmful …, nothing is good about pain at all. If it is not prevented (treated early), it can make a person disabled, can kill, and at the end it can cause loss of assets (excess cost), even for no cure. Generally, pain means harm …” (FGD1 Participant #5, 35yo).

Surprisingly, pain was perceived not only as a symptom of other illnesses but also as a disease by itself since the terms pain (**himem, dhisso or dhukkubbii**, in local languages) and disease (beshita, dhibba or dhukkuba, in local languages) were interchangeably used to describe one another.

### 3.2. Theme 2: Sustenance for Pain Relief

Various natural pain-relieving methods, such as nutrition-related approaches and body–mind relaxing techniques, were commonly used to obtain pain relief. Drinking flaxseed juice, fresh milk, water, coffee, and honey are nutrition-related pain-relieving approaches that were commonly mentioned:

“I drink fresh milk, and cold flaxseed juice which is kept in refrigerator … There is [also] a friend who had pain similar to that of mine, she used to drink flaxseed juice, and there is also another person [I know] who drinks water [to get pain relief]” (cholelithiasis patient, 20yo); and “… I drink water … and get some [pain] relief … (Patient #6, 60yo)”.

It was also revealed that there was the experience of using other food substances for pain relief among other people in the community: “… to get pain relief some people use honey” (Patient #10, 45yo). People drink coffee to treat their headaches, as described by a respondent, “I had a headache … For the same problem of headache, people in my neighborhood drink coffee” (FGD2 Participant #5, 35yo).

In addition to water and other food substances, body and mind relaxing activities were also practiced as a means of pain relief. These activities included taking rest, sleeping, and physical exercises: “[I] Play volleyball, … I feel better if I get pain and (then) play volleyball. Drink water … I move because it gets worse at rest, … I drink water, especially during exercise …” (Patient #9, 28yo). Sleeping and taking rest were also practiced to obtain pain relief: “ I … take rest, rest, only taking rest while my legs remained open, … (and also) chewing Khat [gives me pain relief], but taking rest is my favorite …” (Patient #7, 44yo). Chewing khat (*Catha edulis*)—a famous plant that is grown in parts of East Africa and Ethiopia, and the leaves of which are highly stimulant while chewed– was practiced to facilitate complete relaxation. Contrary to the experience of the aforementioned nephrolithiasis patient, avoiding movements and placing oneself in a sitting position also helped to relieve pain in some situations: “Resting, sitting to avoid pain. (Yes,) I get relief when I sit down …” (Patient #11, 18yo). Regardless of its benefits, spending too much time in bed was believed to worsen the pain rather than relieving it with some conditions: “I sleep for some time if I feel pain, but if I spend the whole day in bed, it (pain) worsens” (Patient #6, 60yo).

### 3.3. Theme 3: Traditional Pain Relievers

Non-herbal and herbal traditional medicine were also part of widely used pain-relieving strategies. From the non-herbal healing practices, religious ones—i.e., prayers to God, Allah, or other gods—were commonly used: “In our culture if there is much disease [pain], elderly men and women pray to their gods” (Patient #2, 75yo), and “Some people who prefer to stay praying rather than going to hospitals. They pray. If God helps them, they get cured” (Patient #9, 28yo). Spiritual healing and Western medicine—one complementing another—were also simultaneously used: “Sometimes God heals me. If He gets me [pain] relief, I [always] pray to God and [occasionally] go to health facilities. That is what I do” (Patient#8, 42yo). In addition to praying to God, Holy water, i.e., water blessed by priests and churches, was also used by Orthodox Christianity followers during times of pain and suffering: “They (other people with pain) … use holy water, … go to church … I [also] drink the Holy water” (Patient #11, 18yo). These religious approaches were commonly practiced for easing both mild and severe pain, depending on their beliefs. On the other hand, Muslim patients also followed similar religious approaches: “… I do salat, make Dua (prayer) and bow before Allah … and get some relief” (Patient #6, 60yo). Along with prayers, khat was also mentioned to be chewed by some Muslim respondents: “I make Dua while chewing Khat” (Patient #7, 44yo). In this context, khat was used as a means of improving the believer’s concentration while praying to Allah, in addition to its benefit as a relaxing substance.

Herbal traditional medicine was also widely used as a pain booster among the patients, families, and other community members: “For the same problem of headache … Some people use [herbal] traditional medicine. For diseases like cancer, treatments at hospitals are not effective and people use traditional medicine for cancer as well as its pain, and it is effective” (FGD2 Participant #2, 26yo).

Although herbal traditional medicine is believed to be effective in most cases, it is not free of drawbacks: “I used to take Habesha medicine [herbal medicine in Amharic language] whenever I get it. That’s how I help myself … Some [traditional medicine] gave me pain relief, some didn’t, even worsened it.”… (Patient #2, 75yo). Furthermore, it was noted that much reliance on traditional medicine had also delayed visiting health facilities and treatment-seeking for pain and other conditions.

### 3.4. Theme 4: Conventional Pain Medicine

Despite the wider use of folk medicine, Western medicine played a role in treating pain among surgical patients and their families. People who tend to use conventional medicine had a relatively better understanding of the possible causes of illnesses for which they sought treatment in formal health facilities: “Abdominal pain is due to amoeba. I had cramps, and I used to go to health centers” (Patient#10, 45yo). Similarly, “I used to go to health centers and they gave me a drug that causes flatulence/fart and I got relief” (Patient#5, 39yo). As part of conventional medicine, specific drugs and procedures were also mentioned as used for treating pain: “I … go to the clinics and get injections of painkillers” … [Patient#6, 60yo]; “… [we use] Pain killers, such as diclofenac injected at private clinics in the city …” (Patient#1, 20yo). People in the community—particularly those from urban areas—had also relied on Western medicine: “They go to the health center [for pain treatment] because it is a town”, where Patient#7, a 44-year-old, also mentioned explaining the experience of others in the community. Here, the reason for visiting the health facility to obtain pain treatment seems to be access to healthcare—i.e., health centers and clinics in small towns are close to their residences. In some cases, the respondents tried to justify their tendency to use Western medicine to treat disease or pain with bible verses and religious thoughts: “There were doctors in the bible stories, e.g., Luk was a doctor. Priests encourage church members to go and get treatment in the hospitals” (Patient #8, 42yo).

Unfortunately, some medicines that need extreme caution and are believed to have potential risks of addiction, such as opioids, have also been utilized to ease pain: “I use … tramadol and other unspecified medicine from the pharmacy” (Patient #9, 28yo). There was also a tendency to purchase other pain medicines from open markets and shops, probably from smugglers; however, these drugs were not hygienic and safe, as stated below:

“[Other] People … they also go to shops and get oral medicines which are stored for a long time [expired], if they get a headache, they may buy drugs wrapped with plastic and kept in the shops [along with other commodities] for more than a year. Therefore, they catch other diseases” (Patient #8, 42yo).

## 4. Discussion

This study explored how surgical patients and families from different ethnocultural backgrounds perceived pain and utilized their treatment options at their homes. All patients underwent abdominal surgeries and had pain as one of the clinical symptoms before arrival at the hospital. These findings, however, did not reflect the total picture of the views in the participants’ respective cultures, given the small sample size employed. The presence of pain as a clinical symptom among the patients was congruent with a body of literature that asserts that intra-abdominal surgical pathologies are associated with physical pain [46,47,48,49,50,51,52], and these groups of patients and their families were also expected to share their real experiences related to these phenomena. These findings revealed that patients and families ascribed different meanings and possible reasons for pain and had used a variety of pain-relieving methods. It is imperative to understand how people from diverse ethnocultural backgrounds perceive and deal with pain at home to customize perioperative pain management approaches in health facilities. According to the findings, the study’s participants had some unique *perceptions of meanings*, *causes*, *and consequences of pain*, and mostly utilized natural approaches such as *sustenance for pain relief* and *traditional pain relievers* with or without *conventional pain medicine*.

Pain was perceived as something harmful and presumably caused by or associated with disease conditions, surgical procedures, some ailments, divine punishment for one’s bad deeds, or the work of an evil spirit. It was also perceived as a disease per se by some of the participants. Understanding how patients and other people perceive the causes and consequences of pain, which has much to do with the psychology of pain, can determine how pain is treated by care providers [53]. Aside from the diverse perceptions of pain, multitudes of pain-relieving methods—summarized under the themes of sustenance for pain, traditional pain medicine, and conventional pain medicine—have been used by the participants at home. Being cognizant of surgical patients’ prior pharmaceutical agents (conventional pain medicine) usage helps care providers to anticipate potential adverse effects [54,55], whereas comprehending the trends of complementary and alternative medicine (CAM) usage among patients (discussed under *sustenance for pain* and *traditional pain medicine* themes) is important for developing a sound anesthetic plan, as well as presenting CAM as an analgesic recipe, e.g., as a part of multimodal noninvasive nonpharmacologic analgesia (MNNA) for the interested patients [56,57].

Pertaining to our findings, various perceptions of the causes and consequences of pain, as well as the methods of treating it, have been reported throughout the literature. Perceiving pain as a disease per se may add to the volume of literature that has overwhelmingly scrutinized the pathologic features of clinical conditions. As an attempt to define pain as a disease, Ronald Melzack related abnormal neurologic mechanisms as a cause of chronic pain, exclusively based on the neuromatrix theory of pain [58]. Both short-lived and persistent pain experiences were mentioned throughout our data. However, chronic and acute pain are believed to share some common sensory, emotional, and psychosocial features, except for their timing and some pathophysiological manifestations. In line with our findings, Kahissay et al. reported that “supernatural, natural and social elements” were believed to cause diseases (and related pain) among the community of north-eastern Ethiopia [59]. Aside from the perceived supernatural and religious factors, biological etiologies such as underlying disease and surgical wounds were also acknowledged as causes of pain by our study’s group of current participants. Lovering, in a collaborative inquiry involving participants working in Saudi Arabia and originally from different parts of the world, also reported that pain was perceived to be caused by the evil eye, witchcraft, and the power of ancestors, and suggested taking these factors into account for effective treatment [60]. Lovering also reported that herbal and non-herbal traditional medicine, faith healing, and narcotics were commonly used among cultural groups of Filipino, Asian, Tswana, Saudi, and Afrikaans backgrounds, to various extents. In favor of our findings, Lewis et al., in an evidence synthesis report, described that the origin and meaning of pain were related to cultural views, spiritual thoughts, and personal experiences, and it is also perceived as ‘punishment for bad deeds’, works of ‘evil spirits’, and related to “karma or fate” by Chinese and Indian people [21]. In a review of prevailing evidence, Dhanani et al. also reported that acupuncture, herbal therapy, massage therapy, hypnosis, *tai chi*, and biofeedback were commonly used as nonpharmacologic treatments for various types of pain across the globe [61]. In Dhanani’s report, the efficacy of each nonpharmacologic pain treatment approach was exhaustively discussed, and the quality of the respective evidence was evaluated. Although acupuncture or other similar traditional procedures were not mentioned by our participants as a pain treatment option, hypnosis and biofeedback methods may be somehow related to sleeping and resting, which were mentioned as pain relief approaches in our findings, which is due to the anticipated similarities in their mechanisms of body relaxing effects. However, the report seemed to favor evidence from predominantly Asian and Western cultures, and gave little or no attention to CAM practices in Africa or other regions. Congruent with our findings, Jemes et al., in a systematic review report, described traditional, conventional, and alternative medicine (TCAM) users of Sub-Saharan African regions as people with lower socioeconomic and educational statuses than non-TCAM users [62].

Some features of the biopsychosocial model were reflected in our findings. The underlying disease conditions and surgical wounds (bio-), evil spirit and divine punishment (-psycho), and culturally defined ailments (-social) as the perceived causes or origins of pain were interpreted with the help of the model. With regard to the treatment options, on the other hand, the conventional pain medicine or Western medicine used in the community was related to the biomedical mechanisms, whereas the use of religious healing practices, such as khat, traditional herbs and foods, etc., for pain relief were explained through the psychosocial sub lens of the BPSM.

### 4.1. Perspective for Clinical Practice

The present study revealed that some surgical patients and their families may believe that the causes of pain are not only inflammatory, nociceptive, or related biomedical mechanisms, rather, they also consider the psychological and sociocultural elements as etiologic factors. Their preferred pain treatment approaches may also not be limited to conventional pain medicine, depending on their previous experiences. Such thoughts and practices may not always be true and work well; however, they may affect perioperative patient communication and pain management. For effective preoperative optimization and postoperative analgesic plans, considering prior pain-related behaviors and perceptions, as well as cultural beliefs and practices of surgical patients and their families, may be important in culturally diverse settings. Promoting cultural competence among care providers and carefully integrating the apparently ignored psychosocial elements into the existing biomedically dominated pain assessment tools and management approaches seems to be paramount.

### 4.2. Strengths and Limitations of the Study

Considering the theoretical framework employed, a deeper reflection of the overall positionality, a selection of participants with personal or family histories of painful disease conditions, the use of different data sources as a means of triangulation, and data collection from three different hospitals of ethnoculturally distinct neighboring regional states can be considered as strengths. However, this study also has some limitations, such as a relatively small sample size, manual coding with potential human error, and nonuse of qualitative research software, which limits data visualization and qualitative design-related subjectivity, which can be considered study limitations. Moreover, the findings of the current study cannot be generalized, mainly due to the smaller sample size and the non-probability sampling technique employed during the qualitative data collection. Although the findings are context-specific and not meant for generalizability due to the qualitative design and smaller sample size, the transferability of the findings to similar LMIC settings is still possible. Furthermore, a closer look at the reflexivity statements in the previous section can help readers identify the potential strengths and limitations of the study while interpreting its findings.

## 5. Conclusions

The patients and their family members who participated in the current study have unique perceptions of the meaning, cause, and consequence of pain. They also practiced a variety of pain-relieving approaches. However, professionals commonly treat patients based on how they define pain and view the causes and consequences of pain, mainly considered through a biomedical lens. Such approaches may fail to fully meet the intended goal of pain management in an ethnoculturally diverse setting, where people perceive pain and its causes differently. Awareness about the nonpharmacologic pain-relieving approaches that patients may have utilized not only avoids the adverse events related to their past use but also helps professionals to selectively combine them with conventional medicine as a part of MNNA. Holistic pain management, therefore, may necessitate integrating the group variations and cultural contexts into daily pain assessment and treatment strategies, in addition to acknowledging the individual and subjective nature of the experience of pain, if supported with strong evidence. Regardless of the strength of evidence, evaluating their effectiveness, psychological expectations, and social acceptance of traditional pain interventions may play a great role in helping patients obtain pain relief. Overconfidence in these traditional views and pain-relieving approaches can have drawbacks, such as delaying treatment-seeking at health facilities as well as worsening the condition, probably due to inappropriate dosage and hygiene-related issues. Although the present study described the views and experiences of culturally diverse patients and families in the context of pain and its treatments, which generally holds true for the study’s participants, the findings can also be used with caution in the settings with similar contexts. However, we recommend a large scale quantitative or mixed-method study that is aimed at assessing the prevalence of certain perceptions of pain, patterns of nonpharmacologic pain treatment options, as well as evaluating the effectiveness of interventions that integrate traditional views and approaches to conventional pain management in a perioperative context to generalize similar findings in the future. Moreover, professionals practicing in the hospitals included in this study, as well as those working in similar contexts elsewhere, should be mindful that patients with similar perceptions and experiences of pain and treatment options could present during their daily perioperative practice.

## Figures and Tables

**Table 1:** Summary of participants’ basic characteristics.

Age Range		17 yrs–60 yrs
Sex	Male (%)	47.8
	Female (%)	52.2
Education status	No formal education (%)	30.4
	Primary school (%)	30.4
	Highschool (%)	13.0
	College diploma (%)	4.3
	First degree (%)	4.3
	Unknown (%)	17.6
Residence	Rural (%)	73.9
	Urban (%)	26.1
Ethnicity	Oromo (%)	17.4
	Sidama (%)	39.1
	Gurage (%)	43.5
Diagnosis *	Cholecystitis (%)	13.0
	Intestinal obstruction (%)	13.0
	Myoma (%)	13.0
	Epigastric hernia (%)	13.0
	Nephrolithiasis (%)	21.7
	Gastric Ca (%)	13.0
	Nonspecific intrabdominal tumor (%)	4.3
	Unknown abdominal disease (%)	9.0

* Is the diagnosis of patients of those whose family members are taking care of.

**Table 2:** Themes and texts are compiled under each theme.

Themes	Examples of Texts Extracted from the Transcript
*Perception of pain meaning, causes and consequences*	- “Now it is the wound and previously it was the tumor …” (Patient#7) - “It is the kidney disease that makes me feel pain, Allah knows why I feel pain. Allah brought it to happen, my son, ask others (more reasons why the pain is felt) …” (Patient #6) - “Allah brought it and Alah can get rid of it. Yes it’s related to that …; They say it is due to ‘Offe’ (in Guragigna, it means something swollen). That causes pain.” (Patient #5) - “It is because of the operation. It is because it was touched (cut with surgical knife), … The pain could be due to these reasons. As my body was touched (cut with surgical knife), that could be the reason …” (Patient #8) - “Some people (in our ethnic group) say it is something related to evil spirit.” (Patient #2) - “… I also believe that pain is caused by God.” … (FGD1 participant#4); “sometimes … it is something (related to) a person’s bad deeds that results in the pain of that person …” (FGD1 Participant #5) - “Pain is assumed to be due to cold.” (Patient #10) - “Pain is harmful …, nothing is good about pain at all. If it is not prevented (treated early), it can make a person disabled, can kill, and at the end it can cause loss of assets (excess cost), even for no cure. Generally, pain means harm …” (Patient #6)
*Sustenance for pain relief*	- “I drink fresh milk, and cold flaxseed juice which is kept in refrigerator … There is a friend who had pain similar to mine, she used to drink flaxseed juice, and there is also another person who drinks water … I drink fresh milk, cold flaxseed juice which is kept in the refrigerator …” (Patient #1) - “I drink water …, I take rest, … I drink water and sleep. I sleep for some time if I feel pain, but if I spend the whole day in bed (sleep for too long) it (pain) worsens …” (Patient #6) - “I move because it (pain) worsens when resting. I drink water especially during exercise … Play volleyball, I feel better if I have pain and (then) play volleyball. Drink water …” (Patient #9) - “Resting, sitting to avoid pain. I get relief when I sit …” (Patient #11) - “I have pain, I have headache and when I feel it while at work, I go home and take rest and get relief …” (FGD1 Participant #5) - “I … take rest, rest, only taking rest while my legs are open, … but taking rest is my favorite …”(Patient #7)
*Traditional pain relievers*	- “In our culture if there is much disease (pain), elderly men and women pray to their gods.” (Patient #2) - “I … And make dua (prayer) otherwise.” (Patient #5) - “… drinks traditional medicine, … Dua while chewing khat …” (Patient #7) - “I do salat, make dua (prayer) and bow before Allah.” (Patient #6) - “People drink juices of herbs (traditional medicine), … some people who prefer to stay praying rather than going to hospitals. They pray. If God helps them, they get cured. Some people also use traditional medicine. They use the TM and … Sometimes God heals me. If He get me relieved, I pray to God … That is what I do …” (Patient #8) - “They use holy water, some use traditional medicine, go to church I drink holy water …”(Patient #11) - “If a child get sick and spent the whole night without sleeping, we say he’s been eaten by “buda” (an ‘evil eye’ that is traditionally believed to cause illness), and there are some stuffs hanged on the child’s neck, there are such things (traditional medicines) and the child would inhale it or have washed with it …” (FGD1 Participant #5) - “… Some people use traditional medicine. For diseases like cancer, treatments in hospitals are not effective, but people use traditional medicine for cancer and its pain, and it is effective.” (FGD2 Participant #1) - “I used to take Habesha medicine [i.e., herbal medicine in local language] whenever I get it. That’s how I help myself … Some [traditional medicine] got me relief, some didn’t or even worsened it …”(Patient #2)
*Conventional pain medicine*	- “Abdominal pain is due to amoeba. I had cramps and I used to go to health centers.” (Patient #10) - “I used to take pain killers at local clinics, …. But no traditional medicine used …” (Patient #3) - “I used to go to health centers and they give me a medicine that causes flatulence/fart and I get relief.” (Patient #5) - “I … go to the clinics and get injections of painkillers.” (Patient #6) - “… Pain killers, which are diclofenac injected at private clinics in the city …” (Patient #20) - “They go to the health center because it is a town” (Patient #7) - “I use … tramadol and other unspecified medicine bought from pharmacy.” (Patient #9) - “(Other) People … they also go to shops and get oral medicines which are stored for a long time [expired], if they get a headache, they may buy drugs wrapped with plastic and kept in the shops [along with other commodities] for more than a year. Therefore, they catch other diseases.” (Patient #8)

## Data Availability

The data presented in this study are available on request from the corresponding author due to restrictions related to institutional policy, which was agreed upon during ethical clearance.

## Supplementary Materials

The following supporting information can be downloaded at: https://www.mdpi.com/article/10.3390/healthcare13101142/s1, Table S1: Standard for Reporting Qualitative Research (SRQR).

## Abbreviations

The following abbreviations are used in this manuscript:

FGDs	Focused group discussions
TCAM	Traditional, conventional, and alternative medicine
MNNA	Multimodal noninvasive nonpharmacologic analgesia
CAM LMIC	Complementary and alternative medicine Low- and middle-income countries
